# Interlaminar Shear Properties of Z-Pinned Carbon Fiber Reinforced Aluminum Matrix Composites by Short-Beam Shear Test

**DOI:** 10.3390/ma11101874

**Published:** 2018-10-01

**Authors:** Sian Wang, Yunhe Zhang, Gaohui Wu

**Affiliations:** 1 College of Mechanical and Electrical Engineering, Northeast Forestry University, Harbin 150040, China; daowsa@nefu.edu.cn; 2 School of Materials Science and Engineering, Harbin Institute of Technology, Harbin 150080, China; wugh@hit.edu.cn

**Keywords:** metal matrix composites, Z-pin reinforcement, delamination, carbon fiber, strengthening mechanisms

## Abstract

This paper presents the effect of through-thickness reinforcement by steel z-pins on the interlaminar shear properties and strengthening mechanisms of carbon fiber reinforced aluminum matrix composites (Cf/Al) with a short beam shear test method. Microstructural analysis reveals that z-pins cause minor microstructural damage including to fiber waviness and aluminum-rich regions, and interface reaction causes a strong interface between the stainless steel pin and the aluminum matrix. Z-pinned Cf/Al composites show reduced apparent interlaminar shear strength due to a change in the failure mode compared to unpinned specimens. The changed failure mode could result from decreased flexural strength due to microstructural damage as well as increased actual interlaminar shear strength. Fracture work is improved significantly with a z-pin diameter. The strong interface allows the deformation resistance of the steel pin to contribute to the crack bridging forces, which greatly enhances the interlaminar shear properties.

## 1. Introduction

Carbon fiber reinforced aluminum matrix composites (Cf/Al) have increasingly been used for a variety of automotive and aerospace applications because of their high specific modulus, high specific strength, high thermal conductivity, and low coefficient of thermal expansion [1,2,3,4,5]. However, Cf/Al composites have been reported to have high residual stress, leading to the separation of the interface and delamination cracks during the forming process [4,5]. Delamination cracks cause relatively low interlaminar properties of Cf/AI composites, despite their excellent in-plane mechanical properties. Composites containing these cracks between laminates would suffer from delamination failures when there are out-of-plane loads, causing the severe decline of mechanical properties and earlier structural failure. Thus, it is significantly crucial to improve the interlaminar shear properties of Cf/AI composites for their future applications.

The z-pinning method has been advocated as a simple and effective method to enhance the delamination resistance of composites [6,7,8]. Many studies [6,7,8,9,10] have demonstrated that z-pins can improve the delamination fracture toughness, interlaminar shear strength, impact damage tolerance, and delamination fatigue resistance of composites. z-pins are also effective at increasing the ultimate strength, fatigue performance, and damage tolerance of bonded composite joints [11,12,13].

There are two types of z-pins: Fibrous z-pin and metal z-pin. The effect of fibrous z-pins, which are typically unidirectional carbon fiber rods, on the interlaminar properties has been studied in detail [6,13,14,15]. The literature shows that the fiber z-pin generates a traction force in the bridging zone that reduces the strain energy at the crack tip, thus improving the delamination toughness. In addition, the carbon fiber z-pin is very effective at transforming the crack growth from fast propagation to stable propagation in polymer matrix composites. Less is known about metallic z-pins and their effects on the interlaminar properties of z-pinned composites. Pingkarawat and Mouritz [16] found that the mode I delamination toughness and fatigue strength of carbon-epoxy composites are related to stiffness, strength and fatigue resistance of the material of the z-pin. Zhang et al. [5] reported that the interlaminar shear strength of Cf/Al composites was enhanced by 70–230% using stainless steel z-pins as reinforcements and found that the interfacial reaction layer between the metal pin and the Al alloy was controlled by the z-pin diameter. However, a detailed investigation on the strengthening mechanism of the metal z-pin was not conducted. Ko et al. [13] found that jagged stainless steel pins can increase the static strength and fatigue strength of composite single-lap joints by 11.8–65.8% in different environments, such as various temperatures and relative humidity. The improvement of the mechanical properties was attributed to the transfer of the fastening force between the reinforcing pin and the matrix material through friction. Although the effectiveness of stainless steel z-pins to enhance the interlaminar properties of composites has been proven, their interlaminar strengthening mechanisms have not been systematically addressed and understood.

Recent studies [14,15,16,17] on delamination fracture mechanisms have focused on observation and analysis of the fractographic results of z-pinned composites using optical microscopy and scanning electron microscopy (SEM). These methods could not work well in comprehensively analyzing the interlaminar strengthening mechanisms of z-pinned composites. As an excellent nondestructive inspection method based on absorption contrast, X-ray radiography is a better choice to observe z-pin’s deforming and composites’ delamination, with z-pin located at their original place in the composites [18,19].

The purpose of this paper is to study the Mode II interlaminar shear properties of Cf/Al composites reinforced with stainless steel z-pins by the short-beam test method. The influence of stainless steel z-pins parameters, including the volume content, diameter, and interval, on the interlaminar shear properties of composites is discussed. X-ray radiography is used to observe deformed specimens and to evaluate the role of the steel pins in the Mode II interlaminar fracture process. This study would enhance the understanding of Mode II interlaminar strengthening mechanisms of z-pinned Cf/Al composites.

## 2. Experimental

### 2.1. Materials

The matrix in this study was a 5056Al alloy purchased from Northern Light Alloy Company Ltd., Harbin, China; it had the following chemical composition (wt.%): Al: 94.89%, Mg: 4.80%, Fe: 0.12%, Mn: 0.07%, Si: 0.06% and Cr: 0.06%. M40 carbon fiber (purchased from Toray Industries Inc., Tokyo, Japan) and AISI 321 (Shanghai Baosteel Group Corporation, Shanghai, China) stainless steel were used as the reinforcing material and the z-pin, respectively. The properties of the M40 fibers, matrix alloy, and AISI 321 are presented in Table 1.

### 2.2. Sample Preparation

**Figure 1:**
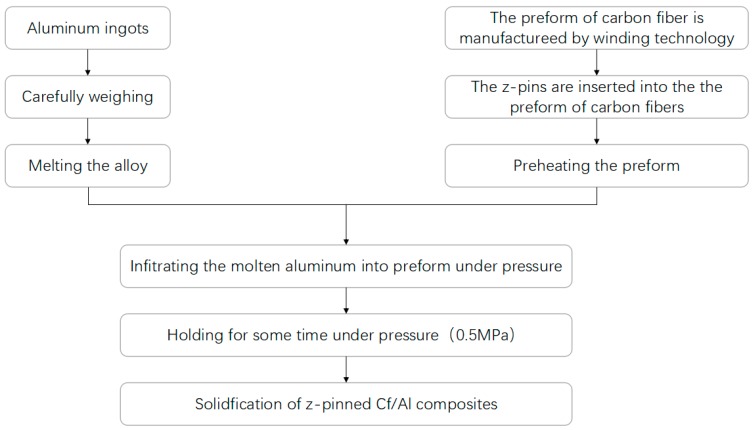
Fabrication procedure of z-pinned Cf/Al composites.

The z-pinned and unpinned Cf/Al composites were fabricated by the pressure infiltration technology. The Cf/Al composite without z-pin reinforcement was produced as the control material to study the changes to the interlaminar properties of the z-pinned composites and the interlaminar strengthening mechanisms of stainless steel pins. The fabrication procedure of investigated composites is shown in Figure 1. Preforms were made using the stainless steel and the carbon fiber, which was unidirectionally winded to the specific shape by a CNC Winding Machine. The preheating temperature of the steel mold with the preform was 500 ± 10 °C. Then the molten 5056Al alloy was infiltrated into the preform under pressure at 780 ± 20 °C, and the infiltration pressure was kept at 0.5 MPa. The composites fabricated were then annealed at 330 °C for 0.5 h.

Several types of z-pinned Cf/Al composite specimens and the unpinned Cf/Al composite specimens were made for a short-beam interlaminar shear test (Table 2). The influence of the z-pin diameter and volume content on the interlaminar properties was studied. Composites were reinforced with stainless steel pins with diameters of 0.3, 0.6, and 0.9 mm and had volume contents of 0.25%, 0.5%, and 1.0%. For the fixed 0.3 mm diameter of z-pins, the volume contents were varied between 0.25%, 0.5%, and 1.0% to study the influence of z-pin volume content on the interlaminar properties. For these 0.3 mm diameter z-pins, the intervals between the pins were 5.3, 3.8, and 2.7 mm. With a fixed 2.0% volume content of z-pins, the z-pins’ diameters were varied between 0.3, 0.6, and 0.9 mm to study the influence of z-pin diameter on the interlaminar properties. The intervals between the pins were 2.7, 5.3, and 8.0 mm for the diameter of 0.3, 0.6, and 0.9 mm, respectively. The volume content of carbon fiber in the unpinned and z-pinned composites was approximately 55%.

### 2.3. Characterization Techniques

**Figure 2:**
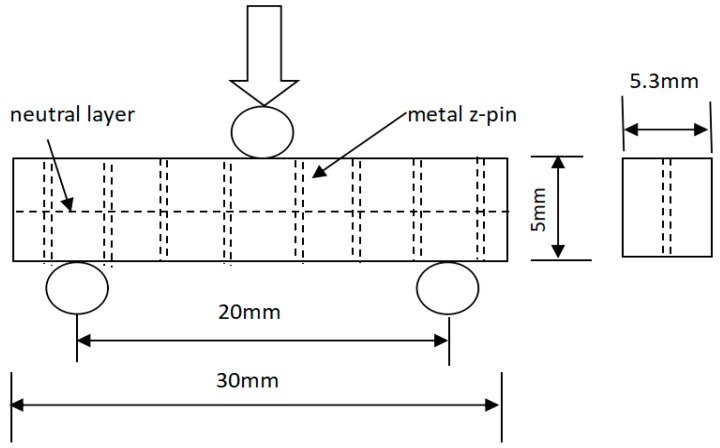
Schematic of short beam shear testing.

The interlaminar shear strength of the unpinned and z-pinned composites was determined using the short beam shear test based on the classical beam theory. The specimens were machined into their dimensions of 30 mm long, 5.3 mm wide and 5 mm thick. The span of the support points was 20 mm (the span length was equivalent to 4 times the thickness). The tests were performed in accordance with ASTM D 2344 [20] at room temperature using an Instron-5569 universal electronic tensile testing machine with a cross-head speed of 0.5 mm/min. The loading setup of the specimens is shown in Figure 2. The specimens were placed on the central position of two cylindrical supports, and a cylindrical head was used to apply a force at the center of the specimens until failure. Cylindrical supports radius and cylindrical head radius are 5 mm and 10 mm, respectively. The apparent interlaminar shear strengths of z-pinned and unpinned composites are calculated using

*τ* = 3*P*/4*bh* (1)

where *τ* is the apparent interlaminar shear strength, *P* is the maximum load, *b* and *h* are the width and thickness of the specimen, respectively. Five specimens of each type of composite were tested, and the interlaminar shear strength was averaged from these five replicates.

The microstructures of the z-pinned Cf/Al composites were characterized by the S-4700 SEM (Royal Dutch Philips Electronics Ltd., Amsterdam, Netherlands). The transformation characteristics of z-pins and failure modes were observed by a SEFT225 X-ray camera (GE Sensing & Inspection Technologies GmbH, Ahrensburg, Germany) to investigate the failure mechanism of the z-pinned Cf/Al composites.

## 3. Results and Discussion

### 3.1. Microstructure

**Figure 3:**
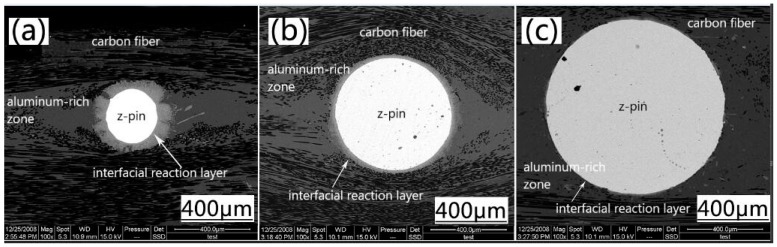
Microstructures of z-pinned Cf/Al composites with different diameters of metal pins (**a**) φ0.3 mm, (**b**) φ0.6 mm and (**c**) φ0.9 mm.

Figure 3 shows the microstructure of z-pinned Cf/Al composites. The interface layer between AISI 321 steel and Cf/Al is clearly visible, indicating a good combination of the two materials. The Cf/Al composites reinforced by different diameters of steel pin show a high denseness without defects such as porosity and cracks. Compared to a Cf/Al composite without a z-pin, a fusiform aluminum-enriched region is formed around the z-pin in z-pinned composites. During the sample preform preparation phase, the z-pin squeezed the surrounding carbon fibers and a void around the z-pin was left; this gap was then filled by AI at a later stage of melt Al being poured into the preform. The matrix enrichment is equivalent to the increase of z-pin diameter to further increase the interlaminar strength of the material. However, the yielding of the fiber could result in a certain angle between the fiber orientation and the direction of the force of the composite material, and thus the decrease of in-plane properties of the laminate including tensile, compressive and bending properties [6].

In addition, Figure 3 also shows that, as the z-pin’s diameter increases, the thickness of the interfacial reaction layer between the steel and the matrix gradually decreases. This is because as the z-pin’s diameter increases, the volume becomes larger, requiring more heat to achieve the same increase in the temperature. But, the contact time between the z-pin and the Al liquid in the sample preparation process is too limited to complete the heat exchange. Within the same period, the z-pin with smaller diameters has a larger temperature increase, stronger atomic diffusion ability, and a greater degree of interface reaction.

### 3.2. Apparent Interlaminar Shear Strength

**Figure 4:**
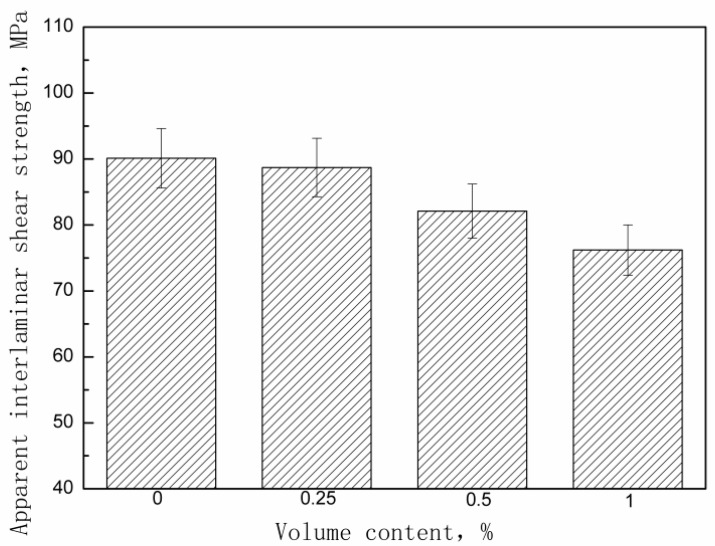
Effect of metal pin content on apparent interlaminar shear strength of z-pinned Cf/Al composites using stainless steel pins with diameters of 0.3 mm.

The effect of the z-pins volume content on the apparent interlaminar shear strength of the Cf/Al composites is shown in Figure 4. The strength decreases with increasing volume content of the stainless steel pins. Table 3 shows the apparent interlaminar shear strength experimental values for each group of z-pinned Cf/Al composites and unpinned Cf/Al composites as well as their standard deviations. The very small standard deviations confirm that adding metal z-pins affects the apparent interlaminar shear strength of Cf/Al composites in a statistically significant way. This is consistent with the results from other researchers. For example, Mouritz et al. [9] reported the similar experimental results in carbon-epoxy composites.

This apparent strength degradation is believed to be related to a change in the fracture mode of the investigated composite with different volumes of stainless steel pins. The failure mode mainly depends on the mechanical properties of the composite and the span-to-thickness ratio of the specimen. We can see that for the composite without z-pins, shear delamination and tensile fracture are caused by the combination of shear and bending stress. The shear delamination is induced by the low interlaminar shear strength of the composites without z-pins. However, for the z-pinned composite, the failure occurred only by fracture along the specimen cross section. The stress state in short beam shear test specimens was complex, involving a combination of compressive, tensile, flexural and interlaminar shear stress. The failure of specimens often occurred by flexural and interlaminar shear stress and stainless steel z-pins were effective in enhancing the actual interlaminar shear strength. Hence, the flexural strength was also the decisive factor for the apparent strength value.

The flexural strength of composites decreases with increasing volume content of the pins typically owing to in-plane fiber waviness and matrix-rich zones caused by the z-pins. The decrease of flexural strength could cause the reduction of the apparent interlaminar shear strength [21].

**Figure 5:**
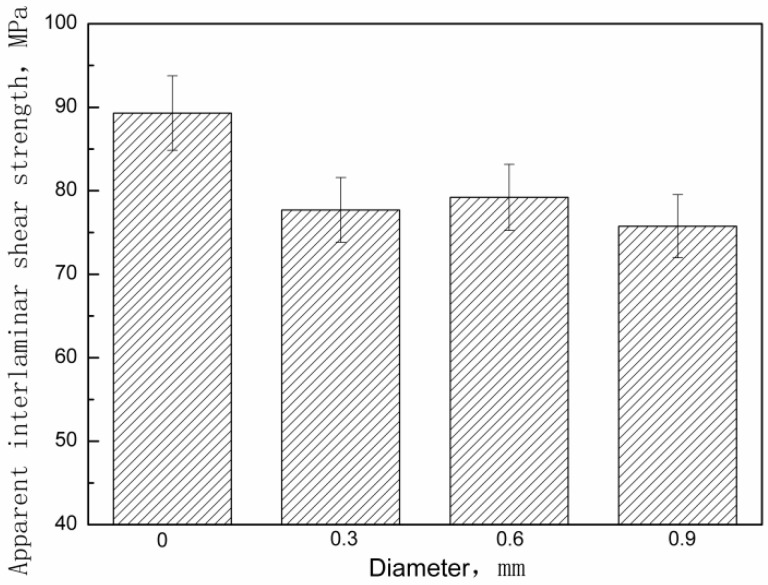
Effect of metal pin diameter on apparent interlaminar shear strength of z-pinned Cf/Al composites using stainless steel pins with a volume content of 1%.

The effect of pin diameter on the apparent interlaminar shear strength of the composite is shown in Figure 5. The apparent strength values are almost the same when the pin size was increased at fixed pin volume content. Table 4 shows the measured apparent interlaminar shear strength for each group of z-pinned Cf/Al composites and unpinned Cf/Al composites as well as their standard deviations. A possible explanation is that increasing the pin diameter may not lead to the decline of the flexural strength of z-pinned metal matrix composites. A further study is being conducted on the mechanism and effects of the steel pins’ diameter on the flexural strength.

### 3.3. Analysis of Stress-Strain Curves of Z-Pinned Cf/Al Composites by the Short-Beam Test

**Figure 6:**
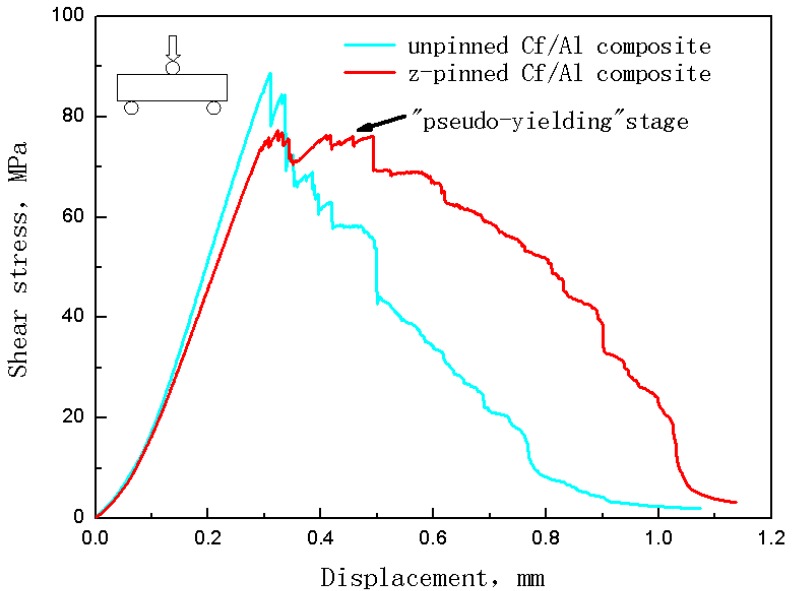
Typical shear stress-deflection curve of Cf/Al composites with and without z-pins.

Figure 6 shows the shear stress-deflection curve of a Cf/Al composite without z-pin and that of a Cf/Al composite with a z-pin volume content of 1% and 0.6 mm diameter (Other the shear stress-deflection curve of the z-pinned composite using stainless steel pins with different parameters look similar). For the unpinned composite at the beginning of loading, the shear stress increased linearly up to the maximum stress where it saw a significant and rapid decrease. The z-pinned composite had a similar elastic deformation stage to that of the unpinned composite. However, after reaching maximum shear stress, the z-pinned composite had a certain amount of deflection where the shear stress only gradually decreased with a small amount of fluctuation. The z-pinned composite in this stage retained a high shear-bearing capacity. The deformation characteristics in this post-maximum shear stress stage are similar to the tensile yield characteristics of metallic materials. Thus, this stage can be regarded as a “pseudo-yielding” stage. Following this stage, the shear stress decreased significantly to final fracture.

**Figure 7:**
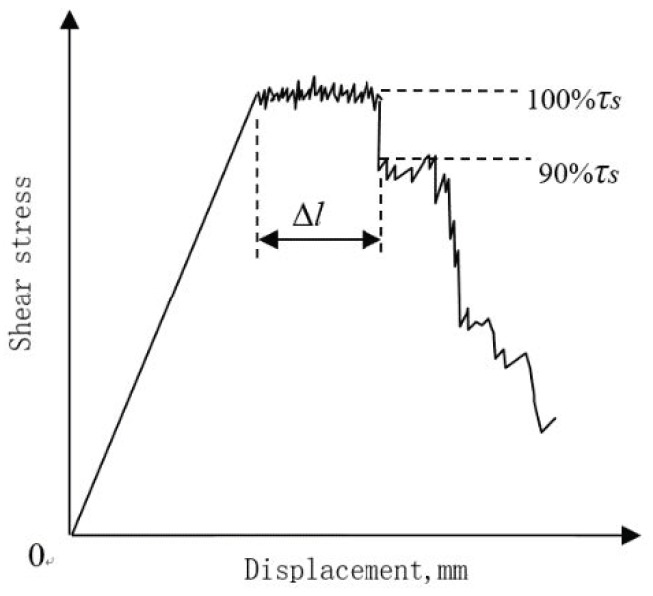
Schematic diagram showing definition of the yield platform length.

**Figure 8:**
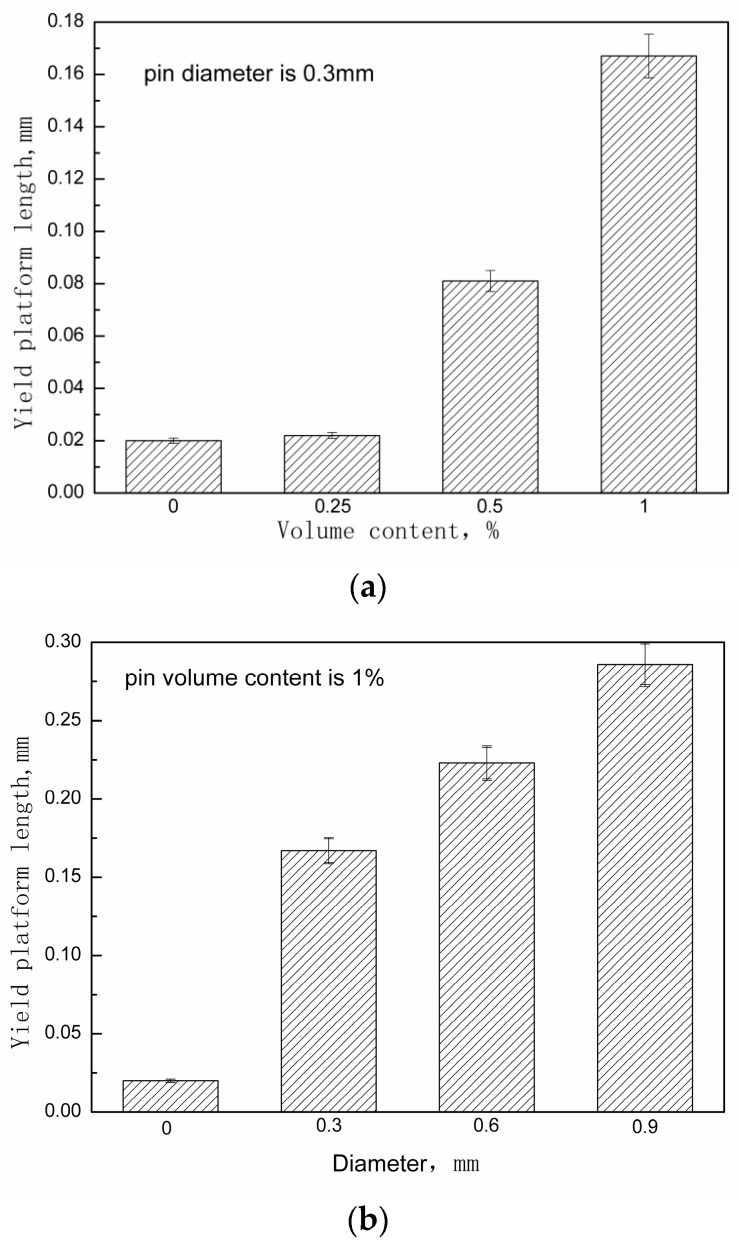
Effect of (**a**) metal pin content and (**b**) metal pin diameter on yield platform length.

**Figure 9:**
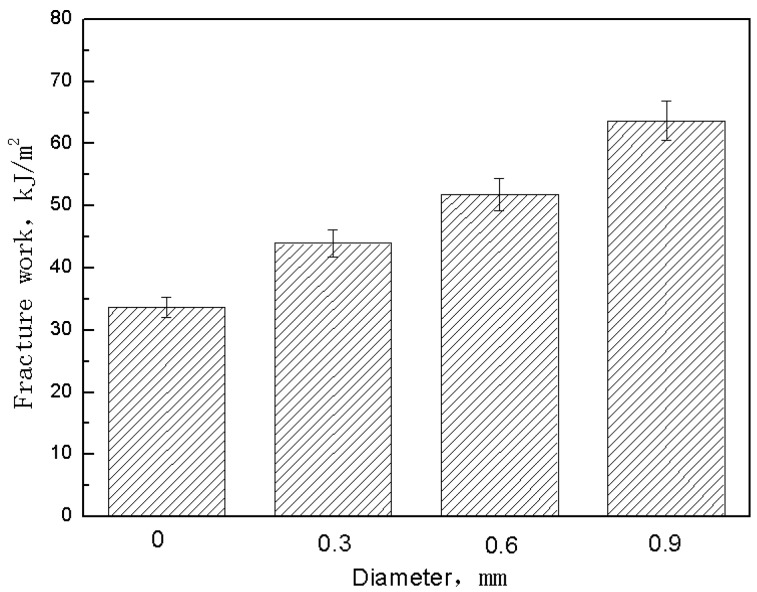
Effect of metal pin diameter on fracture work of z-pinned Cf/Al composites using stainless steel pins with a volume content of 1%.

The steel pin’s geometric parameters’ effect on the extent of deflection in the pseudo-yielding, herein called the yield platform, was quantitatively evaluated. This evaluation of the yield platform defines its length, Δ*l*, as the amount of deflection between the point of maximum shear stress and the point when the stress has decreased to 90% of the maximum value. Figure 7 shows this definition of the yield platform length. The influence of the volume content and diameter of the steel z-pin on the yield platform length is shown in Figure 8. This figure shows that as the volume content of the steel pin and the diameter increased, the length of the yield platform also increased. Table 5 and Table 6 show the measured yield platform length experimental values for each group of z-pinned Cf/Al composites and unpinned Cf/Al composites as well as their standard deviations. In addition, the material maintained a higher load-bearing capacity with longer yield platforms, indicating that the addition of a steel pin may also enhance the interlaminar fracture toughness of the material. This is supported by the effect of the steel z-pin diameter on the fracture work during loading as shown in Figure 9. Table 7 shows the measured fracture work length experimental values for each group of z-pinned Cf/Al composites and unpinned Cf/Al composites as well as their standard deviations. Here, it is seen that increasing the diameter of the steel pin does not increase the shear strength of the short beam; however, it did significantly increase the fracture work. This was attributed mostly to formation of a bridging zone caused z-pins spanning the crack. The crack bridging forces effectively resisted the propagation of delamination cracks and remarkably reduced the opening stress at the crack front. Thus, the fracture work and length of the yield platform could be significantly improved with z-pins. As the bridging forces are transmitted by the interface of the composite and stainless steel pin, improvement to the interlaminar shear property is controlled by the total interfacial contact area between the composite and z-pins. Hence, the length of the yield platform increases with the diameter and volume content of z-pins.

As the z-pinned specimen deflection continued, the stress began to decrease. Compared to the unpinned composite, the pinned composite had a lower rate of decrease of the shear stress prior to failure.

### 3.4. Interlaminar Strengthening Mechanisms

The specimens were examined using X-ray imaging before and after testing. As well, specimens were unloaded at different deflection levels after reaching maximum shear stress to investigate the failure progression.

**Figure 10:**
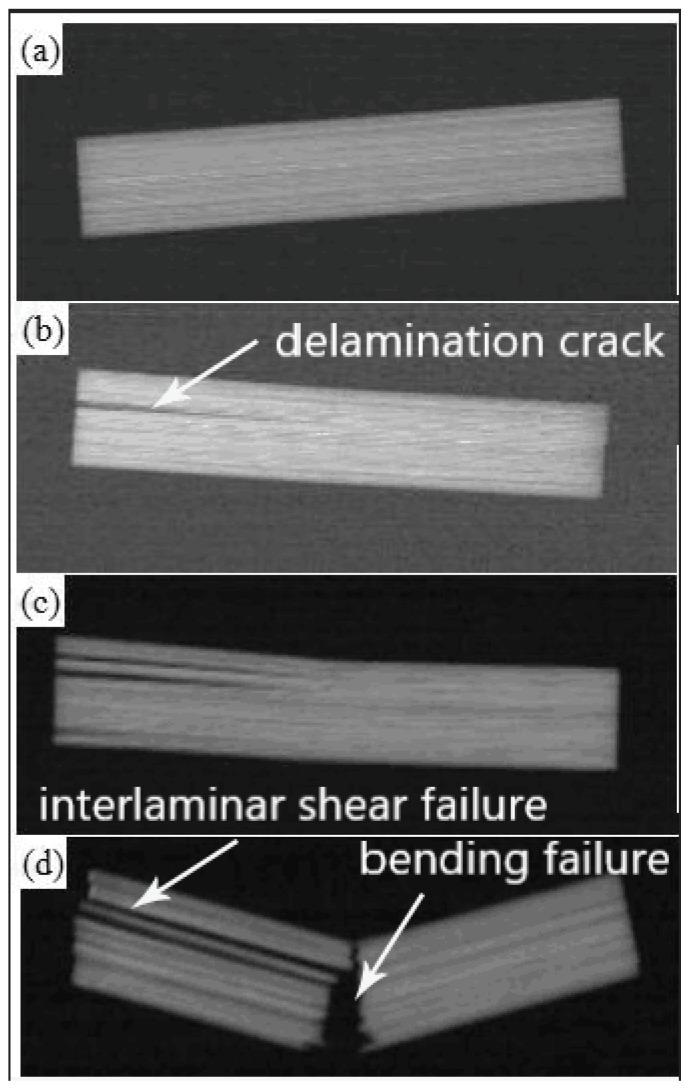
X-ray images of unpinned composite (**a**) before testing, (**b**) at maximum shear stress, (**c**) when load-bearing capacity dropped to 50%, (**d**) after fracture.

The unpinned composite specimen X-ray images are shown in Figure 10. The progression in this figure indicates that a delamination shear failure occurred for this type of specimen. In the early stages of failure (Figure 10b), the unpinned composite had a single delamination crack that initiated from the specimen edge and propagated to the middle of the specimen. In addition, the initially formed delamination crack at maximum shear stress showed unstable crack propagation as it progressed into the middle of the specimen.

When the load-bearing capacity had dropped to 50% of its maximum value (Figure 10c), the unpinned composite specimen had undergone multiple delamination failures. There were no significant tensile or compressive failures observed on the upper and lower surfaces of the specimen. Thus, the failure mode was still delamination shear failure.

The final fracture of the unpinned composite specimen shown in Figure 10d demonstrates the severity of this delamination. It is clear that the delamination shear failure of the specimen becomes more severe with increasing deflection. When the tensile stress on the lower surface of the specimen exceeded the tensile strength of the composite, the specimen had a hybrid failure of shear delamination and tensile fracture.

**Figure 11:**
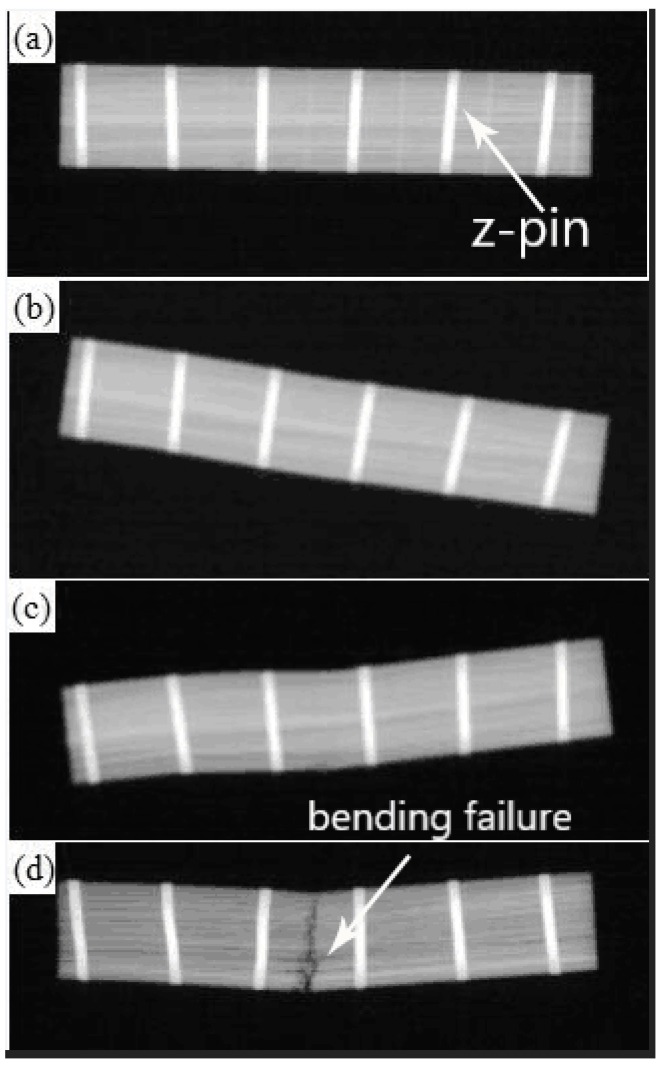
X-ray images of z-pinned composite with a z-pin volume content of 1% and 0.6 mm diameter (**a**) before testing (**b**) at maximum shear stress (**c**) when load-bearing capacity dropped to 50% (**d**) after fracture.

X-ray images of the z-pinned composite with a z-pin volume content of 1% and 0.6 mm diameter are shown in Figure 11 (Other X-ray images of the z-pinned composites using stainless steel pins with different parameters look similar). The X-ray image corresponding to the end of the pseudo-yielding stage is shown in Figure 11b, which shows no visible opening fracture cracks but residual bending deformation. It is also seen that the steel pin did not plastically deform, which means that there was a good bonding at the steel pin-aluminum interface. However, the drop of shear stress here indicates that the initiation of delamination cracking in composites had occurred due to interlayer sliding displacements caused by bending deformation. It also shows that the addition of the steel pin had little effect on the initiation of delamination cracking in composites. On the other hand, metal z-pins effectively suppressed the propagation of delamination cracks. After the delamination crack was initiated, it was strongly pinned by the steel pin and could only locally expand in the section between two steel pins. The limiting of crack propagation resulted in the stable shear stress which was seen in the pseudo-yielding stage. As a result, deformation progressed with a different process compared to the unpinned composite. This reinforcement mechanism is consistent with the one of reinforcing fibers in carbon-epoxy composites.

Figure 11c shows the X-ray image after the specimen reached 50% of the maximum shear stress value. In this image, the specimen still had no visible delamination damage, and the steel pin–aluminum interface remained well bonded with no observable plastic deformation of the steel pin. However, when compared with Figure 11b, the residual bending deformation in the middle of the specimen was greater. At the edge of the specimen, no deformation was observed. The lack of edge deformation demonstrates that the crack in the high-stress zone in the middle of the specimen did not extend to the stress-free zones of the edges. Residual bending deformation resulted from interlaminar movements between multiple sub-layers of composite materials. Under the action of steel pin transferring stress, the maximum shear stress of the neutral plane was distributed to each sub-layer. Consequently, the interlaminar fracture energy was dispersed and absorbed by those sub-layers. Thus, the failure mode of the specimen was transformed from delamination failure on the maximum shear stress surface to microscopic interlaminar shear failure occurring on each sublayer. As a result, the load-bearing capacity of the specimen slowly decreased with each progressive sublayer failure.

The X-ray observation of the pinned composite specimen after fracture (Figure 11d) shows that the specimen still had no obvious delamination fracture. The steel pin had an S-shaped plastic deformation. The specimen was fractured along the cross-section in the middle. There was no delamination cracking even at final fracture, for the pinned composite specimen. Thus, even if there was a layer misalignment in the specimen, the steel pin effectively inhibited the delamination fracture of the specimen due to the strong steel pin–aluminum interface. Under the condition of large shear deformation, the steel pin had seen significant plastic deformation. Figure 11b,c do not display any significant plastic deformation of the steel pin, which means at these points the pin was still behaving elastically. As a result, it is seen that the deformation resistance of the steel pin hindered the shear failure between the sub-layers of the composite material and eventually deformed as a result of this resistance. It is worthwhile mentioning that S-shaped deformation of the steel pins is caused by out-of-plane shear stress i.e., compressive stress and tensile stress as well as shear stress, which is different from the simple shear deformation due to plane stress and plane strain [22]. Due to the steel pin–aluminum interface, no delamination fracture occurred in the sub-layer in which interlayer displacement had occurred for the unpinned composite. When the tensile stress of the lower surface of the specimen exceeded its tensile strength, the specimen immediately underwent tensile fracture along the cross-section, and the stress rapidly decreased.

This is different from the effects reported for fiber z-pin deformation on shear delamination. This result is due to the difference in interfacial bonding strength and z-pin bending stiffness. In some cases, when the fiber z-pin is in the initial stage of loading the interface between the pin and the composite material is completely bonded, an S-shaped elastic deformation could occur which generates a bridging force that hinders delamination. However, the debonding or shear failure may occur at the interface as the load increases because the pin has a weak interface with the matrix. Therefore, the load causing delamination failure largely depends on the frictional pull-out force caused by interfacial friction. The contribution of deformation of the fiber z-pins is so small that many researchers have neglected the z-pins deformation when modeling the effect of fibers on the properties of the material. In this study, due to an interface reaction between the stainless steel z-pin and the aluminum matrix, there is a high degree of interfacial bonding strength. As a result, the steel z-pins can maintain a good interface with the matrix while being deformed. In addition, the steel z-pin maintains a constant bending stiffness during loading. Therefore, the deformation resistance of the steel z-pin can effectively block the interlayer shift. Thus, the z-pin bending enhances the load-bearing capacity of the material and increases the bridging resistance which prevents delamination. It should be noted that when the specimen failure appeared, both shear fracture and pull-out of the z-pins had not occurred. In other words, the carrying capacity of the z-pins was not fully utilized. This is consistent with the analysis in Section 3.2 regarding the effect of the z-pins parameters on the apparent interlaminar shear strength value.

## 4. Conclusions

The aim of this study was to understand the effect of steel z-pin reinforcement on the interlaminar properties of carbon fiber reinforced aluminum matrix composites (Cf/Al). The three-point beam shear test was performed with different z-pin diameters and z-pin volume contents. X-ray radiography was used to observe delamination propagation and deformation of the stainless steel pin.

The apparent interlaminar shear strength of the z-pinned composites is reduced by 1–27% due to the reduction of the flexural strength caused by the in-plane fiber waviness and aluminum-rich zones. The unpinned composites showed a combination of flexure/interlaminar shear in the failure mechanism due to the complex stress state in short beam shear test specimens. Meanwhile, bending failure was usually the dominant failure in the z-pinned composites since the steel pin is significantly effective at resisting the growth of delamination cracks. It should be emphasized that this change of failure mode is caused by the stainless steel pins improvement due to the actual interlaminar shear strength of composites.

In the shear stress and deflection curve, a yield platform similar to that in the metal tension test is observed. The length of the yield platform increases with the size and volume content of z-pins. The appearance of yield platform appears to be a direct result of the increase of crack bridging forces. The bridging force increases along with the increasing of the total size of the surface area of the composite and stainless steel pin. X-ray radiography reveals that S-shaped deformation of z-pins is the major contributor to the bridging force, which is related to the high interfacial bond strength due to the interface reaction between stainless steel and aluminum matrix.

## Figures and Tables

**Table 1:** Basic properties of 5056Al alloy, fibers and z-pins.

Materials	Density (g/cm³)	Tensile Strength (MPa)	Elastic Modulus (GPa)	Elongation to Fracture (%)
5056Al	2.64	314	66.7	16.0
M40	1.76	4410	377.0	1.2
AlSl321	7.85	1905	198.0	2.0

**Table 2:** Short-beam shear interlaminar test matrix of laminate specimens.

z-Pin Volume Content (%)	z-Pin Diameter (mm)	z-Pin Space (mm)	Interlaminar Shear Properties
-	-	-	Yes
0.25	0.3	5.3	Yes
0.5	0.3	3.8	Yes
1	0.3	2.7	Yes
1	0.6	5.3	Yes
1	0.9	8.0	Yes

**Table 3:** Apparent interlaminar shear strength of unpinned Cf/Al composites and z-pinned Cf/Al composites using stainless steel pins with diameters of 0.3 mm measured by short-beam shear test.

Specimen Number	Apparent Interlaminar Shear Strength (MPa)			
	0 vol%	0.25 vol%	0.5 vol%	1 vol%
1	89.3	88.7	82.1	77.7
2	92.3	90.0	86.8	76.2
3	89.9	91.5	90.3	79.8
4	89.1	87.4	78.4	75.6
5	90.4	86.2	83.2	76.9
Average	90.2	88.8	84.2	77.2
Standard deviation	1.15	1.87	4.07	1.46

**Table 4:** Apparent interlaminar shear strength of unpinned Cf/Al composites and z-pinned Cf/Al composites using stainless steel pins with a volume fraction of 1% measured/determined by short-beam shear test.

Specimen Number	Apparent Interlaminar Shear Strength (MPa)			
	d = 0	d = 0.3 mm	d = 0.6 mm	d = 0.9 mm
1	89.3	77.7	79.2	75.7
2	92.3	76.2	73.9	74.4
3	89.9	79.8	78.5	65.7
4	89.1	75.6	80.5	76.3
5	90.4	76.9	79.4	68.6
Average	90.2	77.2	78.3	72.1
Standard deviation	1.15	1.46	2.29	4.23

**Table 5:** Yield platform length of unpinned Cf/Al composites and z-pinned Cf/Al composites using stainless steel pins with diameters of 0.3 mm measured by short-beam shear test.

Specimen Number	Yield Platform Length (mm)			
	0 vol%	0.25 vol%	0.5 vol%	1 vol%
1	0.02	0.022	0.079	0.167
2	0.019	0.025	0.093	0.175
3	0.017	0.018	0.084	0.164
4	0.024	0.02	0.081	0.153
5	0.021	0.024	0.085	0.178
Average	0.02	0.022	0.084	0.167
Standard deviation	0.0023	0.0026	0.0048	0.0088

**Table 6:** Yield platform length of unpinned Cf/Al composites and z-pinned Cf/Al composites using stainless steel pins with a volume fraction of 1% measured/determined by short-beam shear test.

Specimen Number	Yield Platform Length (mm)			
	d = 0	d = 0.3 mm	d = 0.6 mm	d = 0.9 mm
1	0.02	0.167	0.223	0.286
2	0.019	0.175	0.235	0.274
3	0.017	0.164	0.218	0.285
4	0.024	0.153	0.206	0.254
5	0.021	0.178	0.241	0.296
Average	0.02	0.167	0.225	0.279
Standard deviation	0.0023	0.0088	0.0124	0.0143

**Table 7:** Fracture work of unpinned Cf/Al composites and z-pinned Cf/Al composites using stainless steel pins with a volume fraction of 1% measured/determined by short-beam shear test.

Specimen Number	Fracture Work (kJ/m²)			
	d = 0	d = 0.3 mm	d = 0.6 mm	d = 0.9 mm
1	33.7	44.1	51.9	63.7
2	31.9	46.6	53.4	59.2
3	36.4	47.5	54.4	62.8
4	30.6	40.5	55.1	68.5
5	32.8	41.4	50.2	60.3
Average	33.1	44	53	62.9
Standard deviation	1.95	2.76	1.77	3.24
